# Investigation of a possible extended risk haplotype in the *IL23R* region associated with ankylosing spondylitis

**DOI:** 10.1038/gene.2017.5

**Published:** 2017-04-06

**Authors:** A R Roberts, M Vecellio, A Cortes, J C Knight, C J Cohen, B P Wordsworth

**Affiliations:** 1 Nuffield Department of Orthopaedics, Rheumatology and Musculoskeletal Sciences, Botnar Research Centre, University of Oxford, Nuffield Orthopaedic Centre, Oxford, UK; 2 National Institute for Health Research Oxford Comprehensive Biomedical Research Centre, Oxford University National Health Service Foundation Trust, Oxford, UK; 3 Wellcome Trust Centre for Human Genetics, University of Oxford, Oxford, UK; 4 Institute of Health and Biomedical Innovation, Queensland University of Technology, Translational Research Institute, Princess Alexandra Hospital, Brisbane, Australia

## Abstract

The *IL23R* region on chromosome 1 exhibits complex associations with ankylosing spondylitis (AS). We used publicly available epigenomic information and historical genetic association data to identify a putative regulatory element (PRE) in the intergenic region between *IL23R* and *IL12RB2*, which includes two single-nucleotide polymorphisms (SNPs) independently associated with AS—*rs924080* (*P*=2 × 10^−3^) and *rs11578380* (*P*=2 × 10^−4^). In luciferase reporter assays, this PRE showed silencer activity (*P*<0.001). Haplotype and conditional analysis of 4230 historical AS cases and 9700 controls revealed a possible AS-associated extended haplotype, including the PRE and risk variants at three SNPs (*rs11209026*, *rs11209032* and *rs924080*), but excluding the *rs11578380* risk variant. However, the *rs924080* association was absent after conditioning on the primary association with *rs11209032,* which, in contrast, was robust to conditioning on all other AS-associated SNPs in this region (*P*<2 × 10^−8^). The role of this putative silencer on some *IL23R* extended haplotypes therefore remains unclear.

## Introduction

Ankylosing spondylitis (AS) is the archetype of a group of inflammatory disorders, known as spondyloarthropathies, in which inflammation of the spine and sacroiliac joints is prominent.^1^ It is a polygenic condition for which >100 genetic associations have now been reported.^2, 3^ Many of the genes implicated in AS are also involved in overlapping clinical conditions, such as psoriasis and inflammatory bowel disease.^4, 5^ The importance of the interleukin (IL)-23 pathway in AS was initially suggested by the genetic association with the IL-23 receptor (*IL23R*).^6^ Subsequently, IL23R expressing cells have been identified at sites of inflammation (enthuses) in mice with SpA.^7^ The first *IL23R* association with AS to be described was with *rs11209026*, a missense variant in the cytoplasmic tail that affects IL-23 R signalling.^6, 8, 9^ There is also a second independent association with AS in the intergenic region between *IL23R* and *IL12RB2* (encoding the IL12 receptor-specific β chain).^3^ This association is also apparent in inflammatory bowel disease and Behcet disease.^4, 10^

Recently, we identified a regulatory enhancer between *IL23R* and *IL12RB2* that is modulated by the AS-associated single-nucleotide polymorphism (SNP) *rs11209032*, which is in the region of association independent of the primary coding SNP *rs11209026*.^11^ Homozygosity for the risk allele ‘A' at *rs11209032* reduces enhancer activity and increases the proportion of IFN-γ-secreting CD4^+^ (Th1) cells in cases with AS. It is known that genomic regulatory elements can be complex.^12^ In the present study, we therefore address the possibility that there are other regulatory regions or SNPs associated with this second independent AS association signal. We have used publicly available epigenomic data (that is, ENCODE^13^ and Roadmap^14^ projects) to identify additional putative regulatory elements (PREs) overlapping this region of independent genetic association with AS. We have identified a PRE containing two SNPs associated with AS independently of *rs11209026*, and show that it has silencer activity in luciferase reporter assays. We have performed haplotype analysis and sequential conditional analysis to identify the likely causal variants/haplotypes. Our findings suggest that there may be complex haplotypic regulatory influences in this region, but highlight that the primary AS association is with *rs11209032*.

## Results and discussion

**Figure 1:**
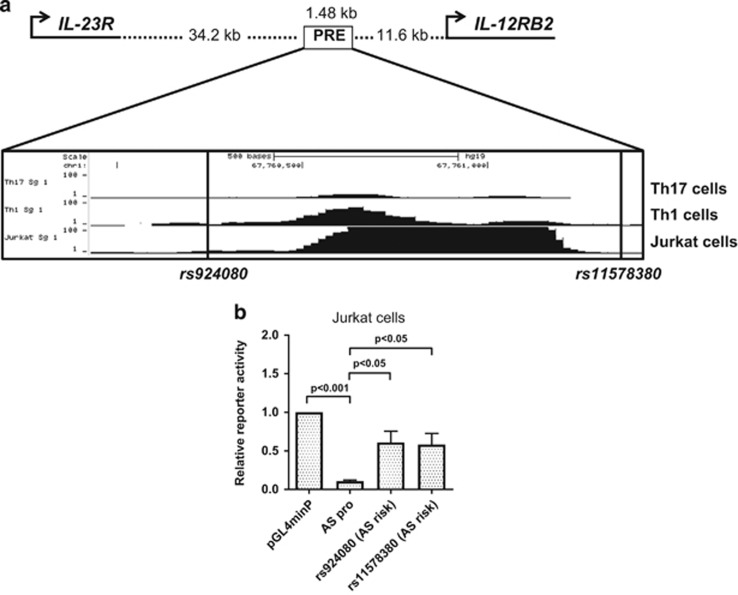
Putative regulatory element containing *rs924080* and *rs11578380* downstream of *IL23R* shows silencer activity. (**a**) Cartoon representation of *IL23R* and *IL12RB2* promoter and putative regulatory element location (Chr1:67,759,931-67,761,417). ENCODE data from UCSC Genome Browser: DNase I hypersensitivity in Th17 cells, Th1 cells and Jurkat cells. (**b**) The transcriptional activity of *rs924080* or *rs11578380* compared to the minimal promoter pGL4minP (set to 1) was measured by luciferase reporter assays in Jurkat cells. The values of relative luciferase activity are expressed as mean±s.e.m. of four repeat experiments each carried out in triplicate. Student's *t*-test was used. minP, minimal promoter.

We first analysed the epigenomic landscape of this region in terms of chromatin accessibility and modifications. This identified a PRE (Chr1:67,759,931-67,761,417) located 34.2 kb downstream of *IL23R* and 11.6 kb upstream of *IL12RB2* (Figure 1a) that overlaps a region of independent association previously reported in AS (conditioned on the primary coding SNP rs11209026).^3^ This region exhibits DNase I hypersensitivity in Th1 and Jurkat cells but not in Th17 cells. Publicly available epigenomic data showed little or no evidence of histone modifications or transcription factor binding,^13, 14^ but these data are only available for a limited range of conditions of cell activation. We then used luciferase reporter assays to investigate the regulatory activity of this PRE in Jurkat cells transfected with pGL4.23 plasmids containing the minimal promoter with or without relevant variants of the 1.48 kb PRE sequence inserted 5′ of this minimal promoter. Transcriptional activity was compared between the minimal promoter construct alone and the wild-type 1.48 kb PRE sequence (AS-protective ‘C variants' at both *rs924080* and *rs11578380*), and also for the *rs924080* AS-risk ‘T' variant and *rs11578380* AS-risk ‘G' variant (Figure 1b). The PRE from the wild-type (protective) variant showed reduced reporter activity significantly below the minimal promoter level (*P*<0.001), suggesting silencer activity. In contrast, the *rs924080* AS-risk ‘T' variant exhibited significantly greater activity than the protective allele (*P*<0.05), which was not significantly less than that associated with the minimal promoter alone. The AS-risk ‘G' variant at *rs11578380* also significantly increased reporter activity to a similar extent (Figure 1b).

To investigate possible regulatory effects arising from multiple SNPs in the *IL23R*–*IL12RB2* intergenic region of association, haplotype analysis was performed using PLINK.^15^ We identified 10 haplotypes for the AS-associated SNPs *rs11209026*, *rs11209032*, *rs6677188*, *rs924080* and *rs11578380* (Table 1). The risk alleles at *rs11209032* (‘A' allele) and *rs924080* (‘T' allele) were always co-inherited on haplotypes 2 and 9 (Table 1). The relatively common haplotype 2 was significantly increased in cases (36% AS vs 32% controls, *P*<2 × 10^−21^), whereas the rare haplotype 9 occurred at a frequency of only 1% in both cases and controls. The protective haplotype 7 was significantly more frequent in controls (4% AS vs 6% controls, *P*<3 × 10^−18^). Together, these data suggest that the risk variants at *rs11209026*, *rs11209032* and *rs924080* might have an additive effect since those associated with AS all appear on the same haplotype (haplotype 2). In contrast, the protective variants at *rs11578380* and *rs6677188* were found on this haplotype, suggesting that these SNPs are unlikely to be involved in any extended haplotypic effect.

To confirm the presence of independent effects from individual SNPs in this putative silencer, we next performed conditional analysis using immunochip data.^3^ The PRE contains three common SNPs (minor allele frequency >1%), two of which previously showed strong association with AS; *rs924080* (*P*=2 × 10^−11^) and *rs11578380* (*P*=1 × 10^−12^).^3^ The associations at *rs924080* (*P*=2 × 10^−3^) and *rs11578380* (*P*=2 × 10^−4^) remained positive after conditioning on *rs11209026*, confirming an association independent of the coding SNP *rs11209026*.^3^ However, this interval of independent association also includes *rs11209032*, as previously reported.^3, 11^ After conditioning on *rs11209032*, the apparent associations at *rs924080* and *rs11578380* disappeared (Table 2). In contrast, the association with *rs11209032* remained positive (*P*<1 × 10^−5^) after conditioning on either *rs924080* or *rs11578380*, thereby confirming *rs11209032* as the primary association with AS in this intergenic region.

These data could also be relevant to Behcet disease with which *rs924080* is strongly associated (*P*<7 × 10^−9^).^10^ Behcet disease is a multisystem inflammatory disorder characterised by symptoms such as uveitis, recurrent oral ulcers, skin lesions and vasculitis; it is also strongly associated with HLA-B*51.^16^ There are occasional reports of the coexistent AS and Behcet disease, but it remains unclear whether this is incidental or overlaps as part of the extended spectrum of spondyloarthropathies, of which AS is the archetype. Patients with coexistent AS and Behcet disease tend to be positive for both HLA-B*27 (AS-associated) and HLA-B*51, suggesting that it may occur incidentally.^17^

In conclusion, we have identified a possible silencer downstream of *IL23R* that includes the AS-associated SNP *rs924080*, which appears to modulate the functional effects of this regulatory element. We have confirmed the primary association of AS with *rs11209032* in this region, but suggest that there could be a possible additional effect from *rs924080* in a putative silencer on the same haplotype. Further work is required to determine the potential functional relevance of *rs924080* to AS and Behcet disease.

## Materials and methods

### Identification of a PRE

We used the published data from genome-wide association studies in AS,^3^ and publicly available data from the ENCODE^13^ and Roadmap Epigenomics Projects,^14^ to identify a 1.48 kb PRE downstream of *IL23R*, which includes two SNPs that are associated with AS—*rs924080* and *rs11578380*. We evaluated DNase I hypersensitivity sites, histone modifications and transcription factor binding-sites previously reported in this region.

### Luciferase reporter assay

The relevant 1.48 kb PRE sequence (Chr1:67,759,931-67,761,417) was amplified from genomic DNA. It was cloned into TA cloning kit pCR2.1 vector (Invitrogen, Paisley, UK) and subcloned into pGL4.23(luc2/minP) reporter vector (Promega, Madison, WI, USA) as previously described.^11^ Point mutations corresponding to genetic variants (T/C) of *rs924080* or (G/C) of *rs11578380* were introduced (primer sequences available on request) using the QuikChange II XL Site-Directed Mutagenesis Kit (Agilent, Santa Clara, CA, USA). Jurkat cells (Clone E6-1, ATCC TIB-152) were cultured in RPMI supplemented with 10% foetal bovine serum, 100 units per ml penicillin, 100 units per ml streptomycin and 2 mm  l-glutamine. A total of 200 000 Jurkat cells per well in 24-well plates were co-transfected with 500 ng of pGL4 construct and 10 ng of pRL-null (Promega) using GeneIn (Amsbio, Abingdon, UK). After 48 h, luciferase activity was measured using the Dual-Luciferase assay reporter system (Promega). Firefly luciferase activity was normalised relative to Renilla luciferase activity for each transfection and calculated as fold increase over pGL4.23(luc2/minP). Four repeat experiments were performed each carried out in triplicate. Student's *t*-test was used to determine statistical significance.

### Haplotype analysis

All cases and controls were of White British ancestry. *IL23R*–*IL12RB2* haplotypes were estimated for 4230 AS cases and 9700 controls from the IGAS immunochip study^3^ using PLINK v1.07. The haplotypes for *rs11209026*, *rs11209032*, *rs6677188*, *rs924080* and *rs11578380* were estimated using the —hap-freq function in PLINK v1.07.^15^ The *χ*^2^-test was used to determine statistical significance.

### Conditional analysis

We used only the same subset of cases and controls of White British ancestry from the IGAS immunochip study to evaluate genetic associations with AS.^3^ Independent effects on genetic susceptibility at the *IL23R*–*IL12RB2* locus were identified by conditional analysis of 4230 AS cases and 9700 matched controls, as previously described.^3^ Association analysis was performed using the logistic regression function in PLINK v1.07, using five principal components to account for population structure.^15^

### Code availability

PLINK v1.07 was used in this study and can be downloaded from https://www.cog-genomics.org/plink2.

## Figures and Tables

**Table 1:** Haplotype frequencies in cases and controls of AS-associated SNPs at *IL23R–IL12RB2* intergenic region

*Haplotypes*	*Immunochip controls (*n=*19 400),* n *(%)*^a^	*Immunochip AS cases (*n=*8460),* n *(%)*^a^	*rs11209026 (risk=G)*	*rs11209032 (risk=A)*	*rs6677188 (risk=A)*	*rs924080 (risk=T)*	*rs11578380 (risk=G)*	*OR*	P*-value*^b^
1	10 (0.05)	6 (0.07)	G	G	A	T	C	1.4	0.5
2	6204 (31.98)	3056 (36.12)	G	A	T	T	C	1.5	<2 × 10^−21^
3	27 (0.14)	6 (0.07)	A	G	T	T	C	0.5	0.1
4	2272 (11.71)	929 (10.98)	G	G	T	T	C	0.9	0.06
5	34 (0.18)	11 (0.13)	A	G	A	C	G	0.7	0.4
6	4887 (25.19)	2047 (24.20)	G	G	A	C	G	0.9	0.3
7	1224 (6.31)	320 (3.78)	A	G	T	C	G	0.6	<3 × 10^−18^
8	2718 (14.01)	1202 (14.21)	G	G	T	C	G	1.0	0.6
9	204 (1.05)	98 (1.16)	G	A	T	T	G	1.1	0.4
10	1774 (9.14)	764 (9.03)	G	G	T	T	G	1.0	0.7

Abbreviations: AS, ankylosing spondylitis; OR, odds ratio; SNP, single-nucleotide polymorphism.

a PLINK analysis for haplotype estimation excludes very rare haplotypes that exist in <0.05% of the population.

b *χ*^2^-test performed on Immunochip cases versus controls.

**Table 2:** Conditional analysis of SNP associations at *IL23R*–*IL12RB2* intergenic region

*Position*^a^	*Conditional SNP*	*Risk/protective*	*SNP*	P	*OR*	*RAF (case/control)*	*LD (*r^*2*^*/D′) with conditional SNP*
Chr1:67706208	*rs11209026*	G/A	*rs11209032*	2 × 10^−8^	1.2	0.96/0.93	0.03/0.97
			*rs6677188*	<3 × 10^−3^	0.9		0.01/0.87
			*rs924080*	2 × 10^−3^	0.9		0.07/0.94
			*rs11578380*	2 × 10^−4^	1.1		0.04/0.94
Chr1:67740342	*rs11209032*	A/G	*rs11209026*	<9 × 10^−14^	0.6	0.37/0.33	0.03/0.97
			*rs6677188*	0.2	1.0		0.17/1
			*rs924080*	0.3	1.0		0.38/1
			*rs11578380*	0.8	1.0		0.5/0.95
Chr1:67760140	*rs924080*	T/C	*rs11209026*	9 × 10^−14^	0.6	0.58/0.54	0.07/0.94
			*rs11209032*	3 × 10^−6^	1.2		0.38/1
			*rs6677188*	0.05	1.1		0.43/1
			*rs11578380*	0.06	1.1		0.61/0.95
Chr1:67761365	*rs11578380*	G/C	*rs11209026*	<4 × 10^−14^	0.6	0.47/0.44	0.04/0.94
			*rs11209032*	1 × 10^−5^	1.2		0.5/0.95
			*rs6677188*	0.3	1.0		0.23/0.89
			*rs924080*	0.1	0.9		0.61/0.95

Abbreviations: Chr., chromosome; LD, linkage disequilibrium; OR, odds ratio; RAF, risk allele frequency; SNP, single-nucleotide polymorphism.

a NCBI Build 37 human genome coordinates.
